# Update on Alcoholic Hepatitis

**DOI:** 10.3390/biom5042978

**Published:** 2015-11-02

**Authors:** Natalie J. Torok

**Affiliations:** 1 Department of Internal Medicine, UC Davis Medical Center, Sacramento, CA 95817, USA; E-Mail: njtorok@ucdavis.edu; Tel.: +1-916-734-3759; 2 Department of Internal Medicine, Northern California VA System, Mather, CA 95655, USA

**Keywords:** steatohepatitis, alcoholic liver injury, oxidative stress, TNFα, IL22

## Abstract

Alcoholic liver disease is one of the most prevalent liver diseases worldwide, and a major cause of morbidity and mortality. Alcoholic hepatitis is a severe form of liver injury in patients with alcohol abuse, can present as an acute on chronic liver failure associated with a rapid decline in liver synthetic function, and consequent increase in mortality. Despite therapy, about 30%–50% of patients with severe alcoholic hepatitis eventually die. The pathogenic pathways that lead to the development of alcoholic hepatitis are complex and involve oxidative stress, gut dysbiosis, and dysregulation of the innate and adaptive immune system with injury to the parenchymal cells and activation of hepatic stellate cells. As accepted treatment approaches are currently limited, a better understanding of the pathophysiology would be required to generate new approaches that improve outcomes. This review focuses on recent advances in the diagnosis, pathogenesis of alcoholic hepatitis and novel treatment strategies.

## 1. Natural History of Alcoholic Liver Disease and Alcoholic Hepatitis

According to the CDC, alcohol is the third leading cause of preventable deaths in the US [1,2], and worldwide it is amongst the leading causes of liver disease. A recent study showed that hospitalizations for alcohol-related events has increased by 25% among the 18- to 24-year-olds in the US between 1999 and 2008 [3], thus, further increase in the incidence of liver disease is expected. Alcohol consumption can induce a wide spectrum of chronic diseases. More than 90% of drinkers develop macrovesicular steatosis, mainly in zone 3. Steatosis is thought to be reversible after cessation of drinking however it can progress to steatohepatitis and fibrosis with continued alcohol consumption. Steatohepatitis is characterized by ballooning degeneration of hepatocytes, Mallory bodies and inflammatory infiltrates consisting mainly of neutrophils. 40%–50% of patients with initial presentation of steatohepatitis progress towards fibrosis and ultimately to cirrhosis within five years of follow up [4]. In women the time to progression to cirrhosis is much shorter than in men, in contrast to HCV and HBV-related liver diseases [5]. Alcohol also contributes to more rapid progression, fibrosis and decline in synthetic function in patients with comorbid conditions such as viral hepatitis, NASH or genetic liver diseases. In a study performed in a large cohort of patients with consecutive biopsies steatosis, fibrosis stage or presence of alcoholic hepatitis at baseline were independent predictors for progression [6]. The genetic predisposition to develop advanced fibrosis has been studied, and polymorphism in patatin-like phospholipase domain-containing protein 3 (PNPLA3) was found to be an important risk factor [7,8,9,10]. This polymorphism may also increase the risk of hepatocellular carcinoma (HCC). Further studies are required to discover environmental and life style differences that can translate into metabolic reprogramming that influence cell fate regulation and alcoholic liver disease outcomes [11].

Alcoholic hepatitis is a distinct, severe form of steatohepatitis that occurs in patients with heavy alcohol drinking [12]. Mortality in patients with severe alcoholic hepatitis is 30%–50% at three months, often despite supportive medical care. The amount of alcohol intake that places a patient at risk of having alcoholic hepatitis is not known. Generally, however, most patients with alcoholic hepatitis consume more than 80–100 g/day of alcohol [13]. Both persistent daily and binge drinking are reported to be harmful with binging defined as intake of ≥5 drinks at a time.

## 2. Clinical Features and Diagnosis

Alcoholic hepatitis is characterized by malaise, fever, sudden increase in bilirubin and/or INR, and complications such as ascites, encephalopathy or variceal bleeding. Serum aminotransferases are elevated with AST > ALT often more than the 2:1 ratio; but AST is usually less than 500 IU/L. Transjugular liver biopsy is recommended, since about 30% of patients can be misdiagnosed based on only clinical parameters. With biopsy potential comorbidities (e.g., viral hepatitis) could also be evaluated. Most biopsies are in fact transjugular but better results are achieved using tru-cut biopsies. Performing tru-cut biopsies revealed that many patients with severe alcoholic hepatitis already can have underlying cirrhosis [14]. A histological scoring system has been developed to facilitate clinical decision making based on neutrophil infiltration, cholestasis, and presence of megamitochondria [15]. To distinguish between alcoholic and nonalcoholic steatohepatitis (NASH), that has similar histological features; the alcohol-non-alcohol index (ANI) can be applied. This formula consists of body mass index (BMI), mean corpuscular volume, AST/ALT ratio, and gender [16].

Obesity and insulin resistance were shown to be independent risk factors for rapid progression of alcoholic hepatitis. Heavy drinkers who are overweight have a higher risk of developing cirrhosis [17]. To *predict disease outcomes*, several models are used. One of the most commonly used model is Maddrey’s discriminant function (DF) that is calculated as 4.6 × (prothrombin time patient − prothrombin time control) + serum bilirubin. A DF value >32 is indicative of potential short-term mortality around 20%–35% at one month [18]. The Model for End-Stage Liver Disease (MELD) also accurately predicts mortality with alcoholic hepatitis [19], with MELD score of 21 having a sensitivity of 75% and a specificity of 75% in predicting 90-day mortality. The age-bilirubin-INR-creatinine (ABIC) score estimates a 90-day risk of mortality, and categorizes patients into low (0%), intermediate (30%), and high (75%) risk of death [20]. The Lille model guides treatment decisions [21] with a score of >0.45 predicting 75% death at six months. Based on the Lille score, corticosteroid treatment can be stopped in those with no improvement after a week of therapy [22].

The presence of systemic inflammatory response syndrome (SIRS) is a major determinant of multiorgan failure and mortality in alcoholic hepatitis. Recently, Michelena *et al.* found that the 90-day mortality was significantly higher among patients with SIRS on admission [23]. Procalcitonin serum levels helped in identifying patients with infection, and lipopolysaccharide (LPS) levels could predict mortality and response to steroids in this study. As corticosteroid therapy can help a subpopulation of patients it is important to screen for infections and determine the risks/benefits of using steroids.

## 3. Pathogenesis

Studying alcoholic hepatitis in the past has been challenging as animal models that follow human pathophysiology have been lacking. Therefore, most of the data were generated and extrapolated from models of chronic alcoholic injury [24]. Recently, the Gao binge model was developed [25] that better approximates clinical conditions with elevation of serum ALT, AST, steatohepatitis with infiltration of neutrophils. This model induces mild injury and is not entirely consistent with alcoholic hepatitis that is observed in patients. It can however be useful to study some pathophysiological features of the disease. To uncover the precise pathomechanism of alcoholic hepatitis and develop new treatment targets, better animal models are still needed.

Ethanol in hepatocytes is oxidized by the cytosolic alcohol dehydrogenase (ADH) to form acetaldehyde. Mitochondrial aldehyde dehydrogenases (ALDH2) then further catalyzes the production of acetate. Acetaldehyde is highly toxic, playing an important role in adduct formation impairing hepatocyte secretory pathways [26], contributing to immune responses [27] and release of inflammatory cytokines [28]. Acetaldehyde and aldehydes can induce collagen synthesis by activation of transforming growth factor β (TGFβ)-dependent and independent profibrogenic pathways and activate hepatic stellate cells (HSCs) [29] leading to progressive fibrosis. Induction of the cytochrome P450 2E1 (CYP2E1) is also a key response to alcohol intake resulting in an increased production of reactive oxidative species (ROS), mainly H_2_O_2_ and superoxide anion [30]. In addition, Kupffer cells [31] and infiltrating neutrophils (through NADPH oxidase 2) are also important sources of ROS. Chronic alcohol exposure results in glutathione depletion (especially in the reduced form) accelerating the effects of ROS [32], leading to endoplasmic reticulum (ER) stress and cell death. In CYP2E1^−/−^ mice [33] or by inhibition of CYP2E1 with clomethiazole alcoholic injury was prevented. However the clinical use of clomethiazole is limited because of potential of causing dependence. Studies using various antioxidants failed to demonstrate a clear benefit in alcoholic hepatitis with the potential exception of N-acetylcysteine (NAC) that reduced the rate of infections and hepatorenal syndrome in patients who were also treated with corticosteroids [34].

Alcohol causes significant changes in the gut microbiota resulting in an altered balance of pathogenic and commensal organisms [35]. As the intestinal mucosal barrier becomes disrupted in alcoholic patients, LPS from gram-negative bacteria can reach the liver and significantly contribute to inflammatory and fibrogenic processes. Recently, gut dysbiosis was shown to induce tumor necrosis factor alpha receptor I (TNFRI) signaling in intestinal epithelial cells that in turn, resulted in the disruption of the intestinal barrier. Although these studies were performed in the chronic Lieber-deCarli model, they may have bearing on the etiopathogenesis of alcoholic hepatitis [36]. In the liver the activation of Kupffer cells by LPS enhances toll like receptor 4 (TLR4) signaling and secretion of pro-inflammatory cytokines, such as IL-1, IL-6 and TNFα, which act on surrounding hepatocytes and stellate cells, as well as elicit adaptive immune responses. In several studies selective intestinal decontamination with antibiotics or prebiotics has shown to reduce plasma endotoxin levels and to prevent liver injury in animal models [35,37]. CD14 a co-receptor for LPS also plays a significant role as CD14^−/−^ mice were resistant to alcohol-induced liver injury [38].

Several studies have established that both innate and adaptive immune responses are dysregulated in alcoholic hepatitis. Recently, investigators found a predominance of proinflammatory Th1 responses in alcoholic hepatitis compared to alcoholic cirrhosis [39]. Further studies therefore are required to develop targeted therapy to inhibit Th1 responses. It also has been observed that antibacterial immune responses are impaired in patients with alcoholic hepatitis. Markwick *et al.* have shown that lymphocytes from patients with alcoholic hepatitis express high levels of immune inhibitory receptors Programmed Cell Death 1 (PD1), and the T-cell immunoglobulin and Mucin Domain-containing Protein 3 (TIM3). As a result the levels of interferon gamma (IFNγ) were decreased whereas IL-10 production increased in response to endotoxins [40]. Targeting these receptors therefore could be a novel strategy to treat alcoholic hepatitis.

Regeneration of the hepatic parenchyma is a key to improve synthetic function. Impaired maturation of hepatic progenitor cells (HPCs) has been demonstrated and was found to be associated with poor prognosis in alcoholic hepatitis patients [41]. Hepatocytes of patients not responding to therapy were characterized by profound mitochondrial damage and lack of cytokines that would be involved in regeneration [42]. In addition there was a significant accumulation of HPCs that differentiated mainly into biliary cells, not hepatocytes. Therefore, methods improving differentiation towards the hepatocytic lineage could be of major benefit.

## 4. Current Treatment Approaches and Potential Therapeutic Targets

Abstinence and correcting nutritional deficiencies are cornerstones of treatment of patients with alcoholic hepatitis. Even moderate alcohol consumption can significantly worsen hepatic hemodynamics, and increase the rate of complications such as ascites and variceal bleeding. Major society guidelines recommend regular assessment of patients for nutritional, vitamin, and mineral deficiencies [43,44].

Patients with DF < 32 have only 10% mortality at 28 days without corticosteroid treatment. Supportive management therefore is adequate for such patients [45]. For patients with severe alcoholic hepatitis the use of corticosteroids is recommended provided there are no absolute contraindications. Corticosteroids were first demonstrated to be beneficial in the treatment of severe alcoholic hepatitis more than 30 years ago. Steroids have a potent immunosuppressant effect with also suppressing pro-inflammatory transcription factors nuclear factor kappa-light-chain-enhancer of activated B cells (NF-κB) and activator protein 1 (AP-1) resulting in lower levels of TNFα and IL-8. However, by applying the Lille model there is a significant proportion of patients who do not respond to steroid treatment with a significantly worse outcome. Because of these patients and the inherent risks of corticosteroid treatment, other therapeutic options are urgently needed. Pentoxifylline is a phosphodiesterase inhibitor, with some effect on lowering TNFα. Although the exact mechanism of action of pentoxifylline in alcoholic hepatitis is not clear several trials have shown a survival benefit, primarily through a reduction in mortality due to hepatorenal syndrome [46]. A multicenter randomized placebo-controlled trial of prednisolone, pentoxifylline, or combination was completed [47]. This showed that four-week treatment with combined therapy compared with prednisolone alone, did not improve six-month survival. In the recently completed STOPAH Trial pentoxifylline did not improve survival whereas prednisolone was associated with a reduction in 28-day mortality that did not reach significance. There was no improvement in outcomes at 90 days or at one year [48].

As for potential future approaches, interleukin 22 (IL-22), a hepatoprotective cytokine, shows promise although most data are from mouse models [49,50]. IL-22 is produced by activated TH17, TH22, and γδT cells, but its receptor is present mainly on hepatocytes. A study in a chronic binge ethanol-fed mouse model showed that treatment with recombinant IL-22 improved liver injury and hepatic oxidative stress while no significant side effects were observed [49]. On the other hand, IL-17 produced by TH17 cells is involved in inducing inflammation. The number of IL-17+ cells is significantly increased in alcoholic liver disease. Therefore a strategy of blocking IL-17 in conjunction with augmenting IL-22 could provide a more efficient method in treating alcoholic hepatitis [51].

Other molecular targets include IL-1 that was shown to have a significant role in alcoholic injury in mice [52,53]. Anakinra, an interleukin 1 receptor antagonist is being evaluated in a clinical trial for severe alcoholic hepatitis (defined as a MELD score ≥ 21). The patients are randomized to receive either methylprednisolone or a combination of anakinra, pentoxifylline, and zinc (ClinicalTrials.gov, AH/NCT01809132). Another trial is studying Rilonacept in alcoholic hepatitis (AH/NCT01903798). As apoptosis or necroptosis are important features of alcoholic hepatitis, Emricasan, an orally active caspase protease inhibitor, is being tested in a phase 2 clinical trial in patients with severe alcoholic hepatitis (NCT01912404).

Salvage liver transplantation in highly selected patients with severe alcoholic hepatitis has been shown to improve survival significantly [54]. Currently, however, a minimum of six months of abstinence from alcohol use and Alcoholic Anonymous (AA) attendance are required for patients with alcoholic liver disease therefore this approach is not available at most transplant centers. Careful studies on the potential of recidivism with a multidisciplinary approach would be required to further evaluate this option.

## 5. Conclusions and Future Perspectives

There is an urgent need to develop unifying criteria and algorithms to diagnose alcoholic hepatitis. As there are several other clinical entities that can have similar presentation; more consideration to liver biopsies should be given to guide treatment decisions. Developing serum biomarkers would facilitate the screening of patients who are at risk for alcoholic hepatitis. Future studies should target patients that have similar outcomes to better delineate factors that may play a role in response to steroids *vs.* partial or no response. Treatment strategies have to be broadened with new, more potent drugs approved and therapies individualized. To achieve this it is essential to develop better animal models. Reversing innate immune responses, limiting inflammatory cascades, improving regeneration and reversing gut dysbiosis could potentially lead to successful combined treatment approaches.
